# Supplementary material for: Molecular elucidating of an unusual growth mechanism for polycyclic aromatic hydrocarbons in confined space
Source: Nat Commun. 2020 Feb 26;11:1079. doi: 10.1038/s41467-020-14493-9 (PMC7044299; doi:10.1038/s41467-020-14493-9)
Supplement: Supplementary file 1 — Supplementary Information [file 41467_2020_14493_MOESM1_ESM.pdf]

Supplementary Information

Molecular elucidating of an unusual growth mechanism for polycyclic aromatic hydrocarbons in confined space

Wang *et al.*

Table of Contents

Supplementary Fig. 1 Photographs of layered SAPO-34 catalyst bed…………………………..... 3

Supplementary Fig. 2 Physicochemical properties of SAPO-34………………………………. 4

Supplementary Fig. 3 The proportion of soluble "coke" and insoluble coke with TOS………..5

Supplementary Fig. 4 Adsorption energy of aromatic species in SAPO-34……………………6

Supplementary Fig. 5 The correlation between micropore volume and "coke" content with "coke" deposition in SAPO-34 for MTO reaction….…………………………….………………….. 7

Supplementary Fig. 6 Direct MALDI FT-ICR MS analysis without framework digestion of SAPO-34 samples with different reaction/deactivating degrees……………………….……………..8

Supplementary Fig. 7 Control experiments for determining the optimal MS parameters….…….9

Supplementary Fig. 8 The photograph and SEM image of the isolated coke……………....….10

Supplementary Fig. 9 The configurational optimization for our proposed cage-passing PAHs…11

Supplementary Fig. 10 MALDI FT-ICR mass spectrum of the extracts and isolated coke…......12

Supplementary Fig. 11 ^{13}C ssNMR spectroscopy of the isolated coke……………..……...13

Supplementary Fig. 12 SEM images and XRD patterns for SAPO-35, SAPO-18 and DNL-6....14

Supplementary Fig. 13 MTO reaction performances over SAPO-35, SAPO-18 and DNL-6…..15

Supplementary Fig. 14 GC-MS analysis of soluble "coke" from spent SAPO-35, SAPO-18 and DNL-6……………………………………………………..……………………………16

Supplementary Fig. 15 Proposal of a complete deactivating model……………..……………...17

Supplementary Fig. 16 Normalized weight loss for the used SAPO-34 for different durations...18

Supplementary Fig. 17 Calibration curves for different aromatic species.…………………….……..19

Supplementary Table 1 Pore textural properties of parent and used SAPO-34…………..…..... 20

Supplementary Table 2 Elemental composition of SAPO-34……………………….…….….21

Supplementary Table 3 Molecular composition of the possible cage-passing PAHs identified...22

Supplementary Table 4 The structural features and the acidity properties of SAPO-35, SAPO-18 and DNL-6………………………………………………………………………….......23

Supplementary Notes……………………………………………………………………….24

Supplementary Figures

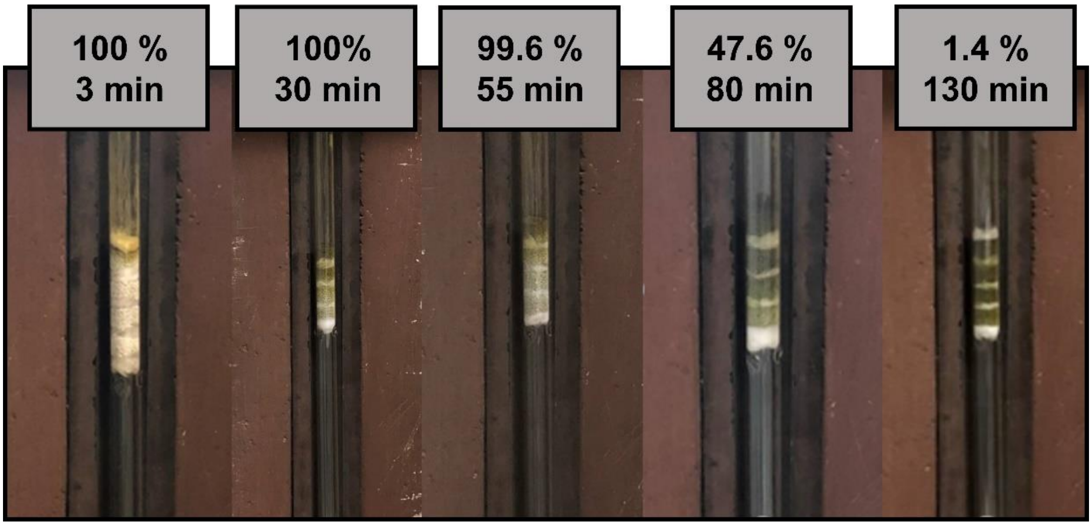

Supplementary Fig. 1 Photographs of SAPO-34 catalyst bed that was equally separated into four layers using quartz wool after MTO reaction at 475 °C with WHSV of 4 h^{-1} for different reaction durations with gradual decreasing conversion. At initial reaction stage of 5 min, only top layer displaying yellow worked as main reaction zone; with reaction proceeding, the catalysts in top layer gradually turned gray and then black, and meanwhile the reaction zone migrated downward.

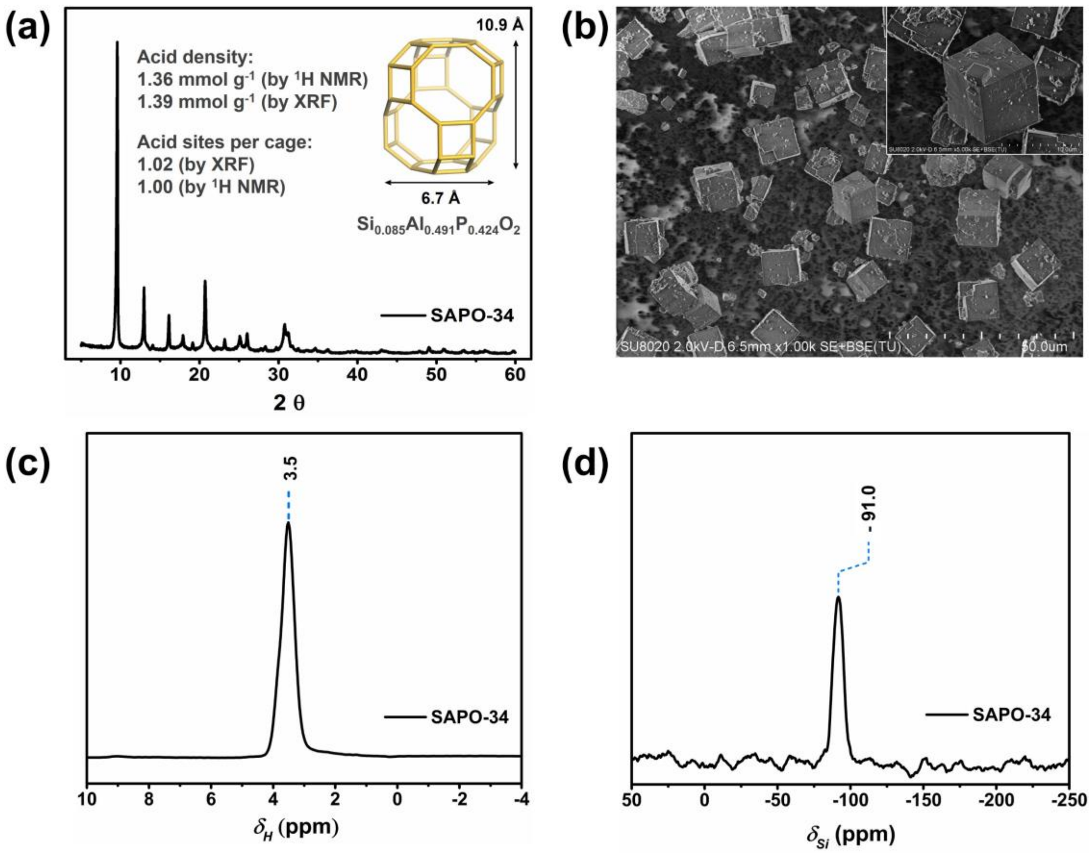

Supplementary Fig. 2 The physicochemical properties of SAPO-34 catalyst. **a**, XRD pattern of synthesized SAPO-34 sample. **b**, Crystal morphology determined by SEM. SAPO-34 exhibits cubic morphology with average size of approximately 6-8 μm. **c**, ^{1}H MAS NMR spectrum of H-SAPO-34. The signal at 3.5 ppm is assigned to bridged hydroxyl group. The amount of BAS determined by ^{1}H NMR spectrum is 1.36 mmol g^{-1} taking adamantane as internal standard, close to the bulky BAS density (1.39 mmol g^{-1}) determined by XRF analysis (shown in Supplementary Table 2). With the determined BAS density, acid site per *cha* cage is estimated to around 1. **d**, ^{29}Si MAS NMR spectrum of hydrated H-SAPO-34. The signals at -91 ppm are assigned to hydrated Si(4Al), indicating the prevalence of Si(4Al) environment.

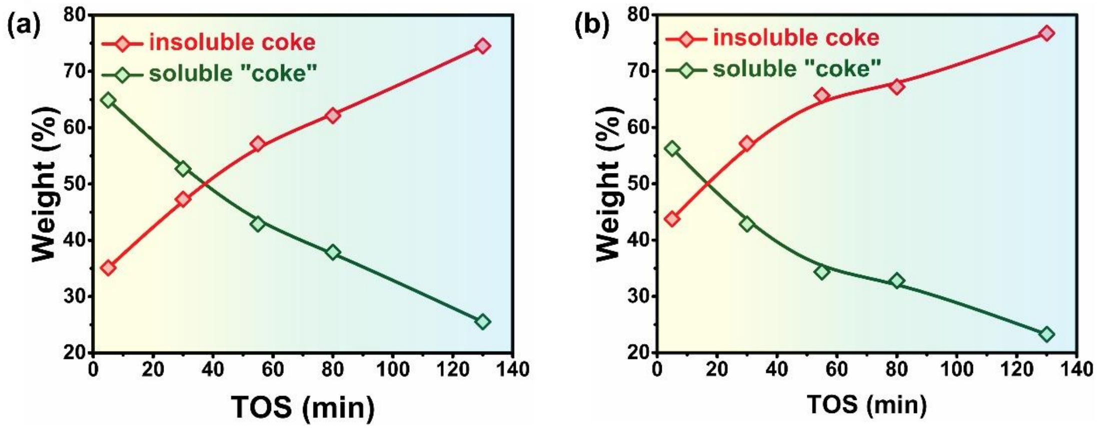

Supplementary Fig. 3 The proportion of soluble “coke” and insoluble coke with TOS by analyzing the samples in the bottom layer of catalyst bed (**a**) and by random sampling of the totally mixed samples of all four layers (**b**).

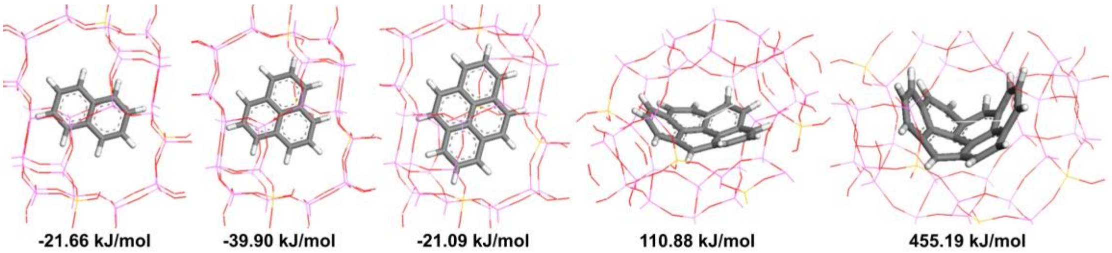

Supplementary Fig. 4 The DFT-derived adsorption energy and optimized molecular conformation of aromatic species with rings ranging from two to six on SAPO-34.

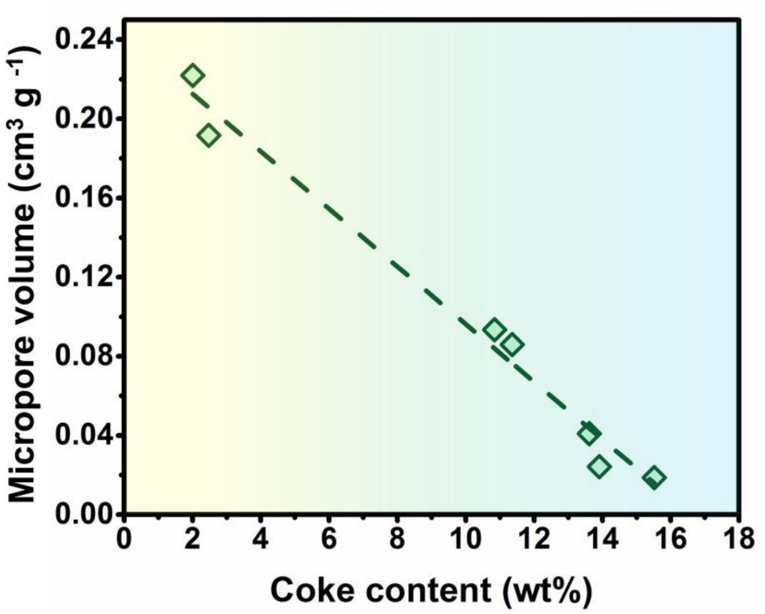

Supplementary Fig. 5 The correlation between micropore volume and “coke” content with “coke” deposition in SAPO-34 for MTO reaction.

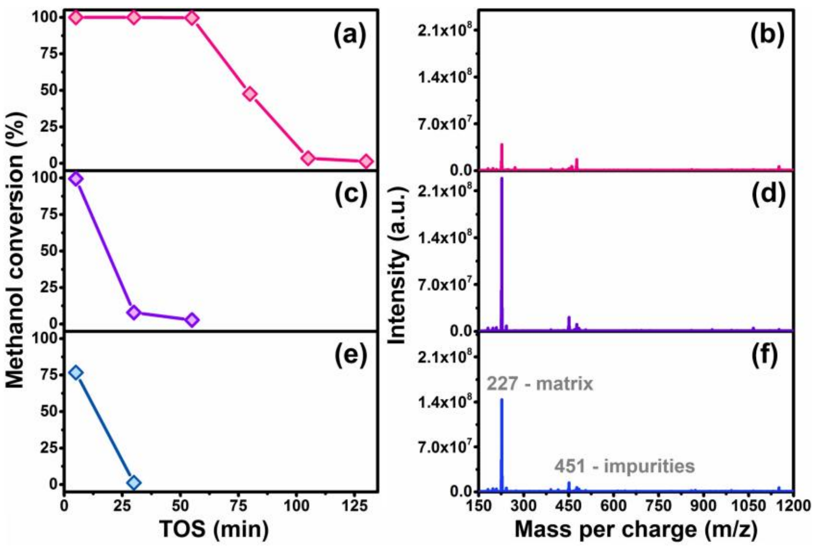

Supplementary Fig. 6 Methanol conversion to obtain spent SAPO-34 samples with different reaction/deactivating degrees and the corresponding MALDI FT-ICR mass spectra of that were directly measured for the used catalysts without framework digestion by HF. **a**,**c**,**e**, MTO reactions under different conditions with temperature of 475 °C and WHSV of 4 h^{-1} (**a**), temperature of 350 °C and WHSV of 13.93 h^{-1} (**b**) and temperature of 350 °C and WHSV of 46.44 h^{-1} (**c**). **b**,**d**,**f**, The corresponding MALDI FT-ICR mass spectra of the direct measurements of the used catalysts.

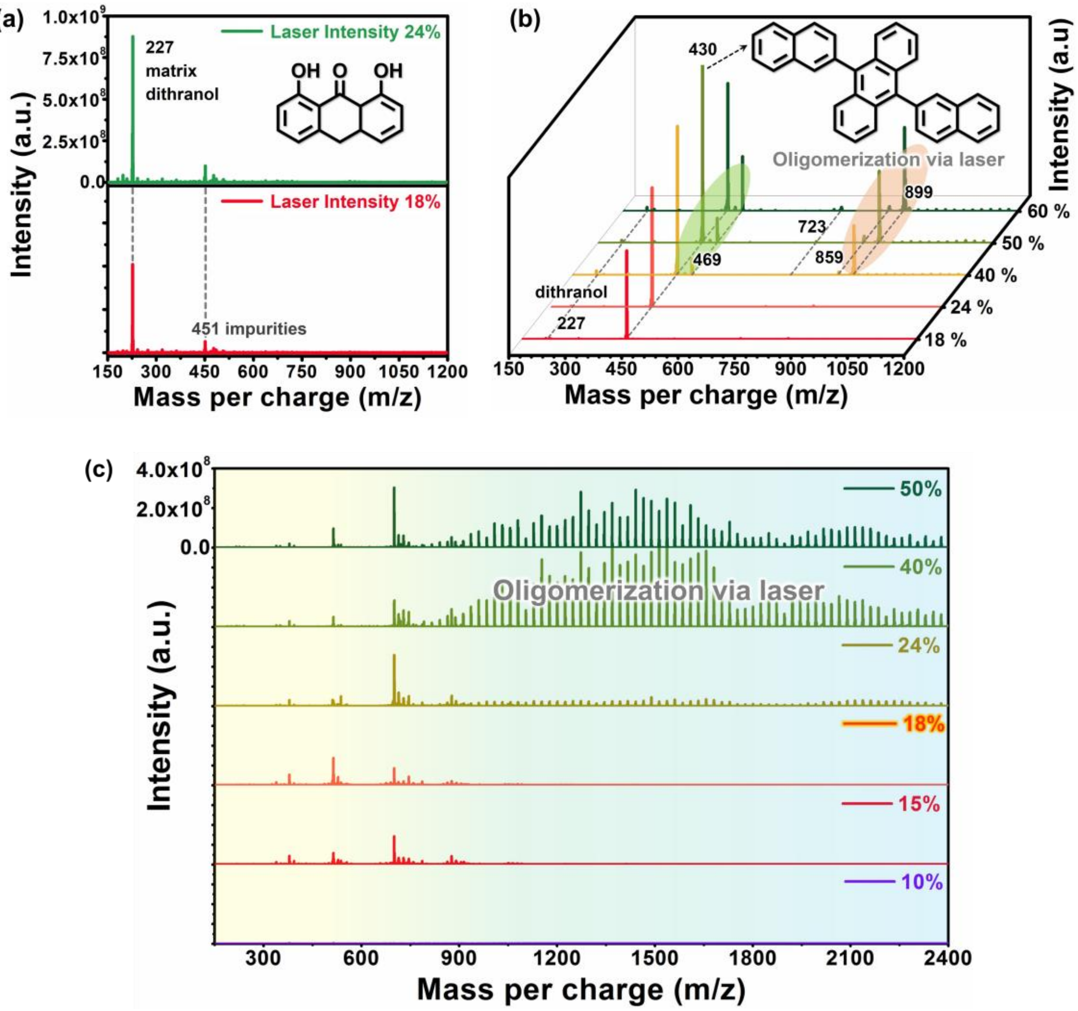

Supplementary Fig. 7 Serious of control experiments for determining the optimal MS parameters. MALDI FT-ICR mass spectrum of matrix (dithranol) **(a)**, model compound (9,10-di-(2-naphthyl) anthracene) **(b)** and extracted coke species from deactivated SAPO-34 after 130 min MTO reaction **(c)** as a function of output percent of laser power for ionization. Mass fragments of 227 Da is assigned to dithranol while the mass range of 451 to 507 Da are attributed to the impurities.

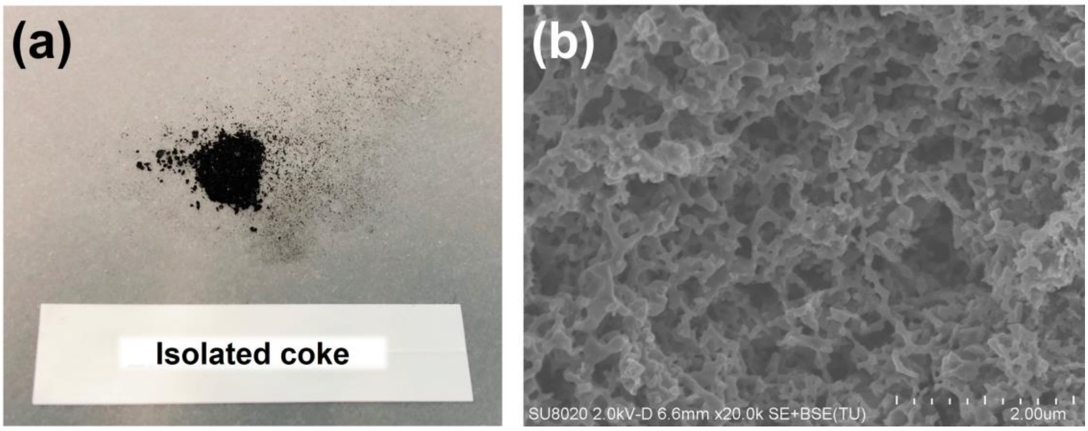

Supplementary Fig. 8 The photograph of isolated coke obtained from the spent SAPO-34 after 130 min MTO reaction by suction filtration of extracts after HF dissolution of catalyst framework (**a**) and SEM image of the isolated coke (**b**).

Model 1

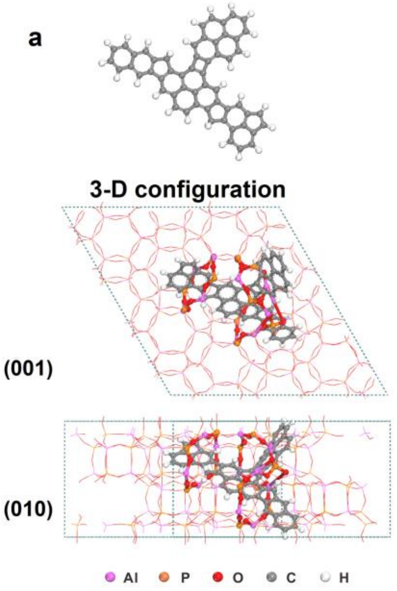

	kcal/mol
E_{inter}	-171.34
$E_{Strain\text{-}zeo}$	105.88
$E_{Strain\text{-}coke}$	56.21
E_{ads}	-9.25

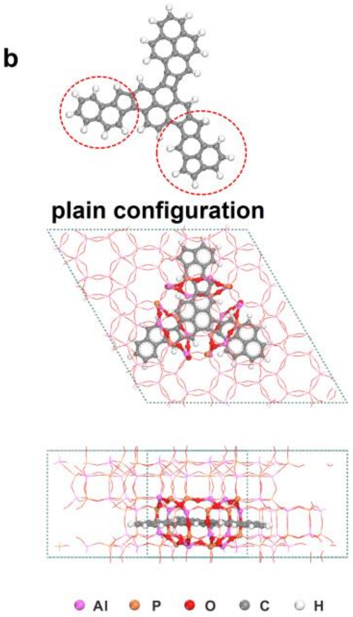

	kcal/mol
E_{inter}	-63.08
$E_{Strain\text{-}zeo}$	33.71
$E_{Strain\text{-}coke}$	50.16
E_{ads}	20.79

Model 2

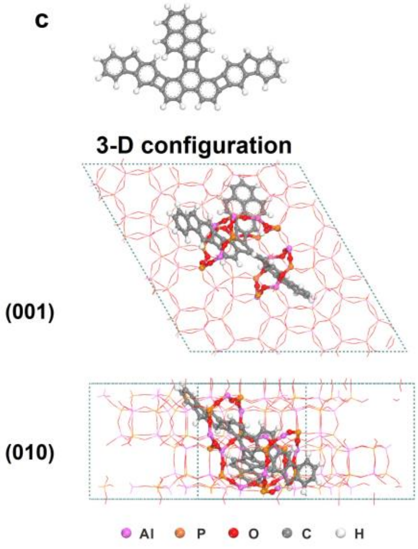

	kcal/mol
E_{inter}	-84.80
$E_{Strain\text{-}zeo}$	2.76
$E_{Strain\text{-}coke}$	74.92
E_{ads}	-7.12

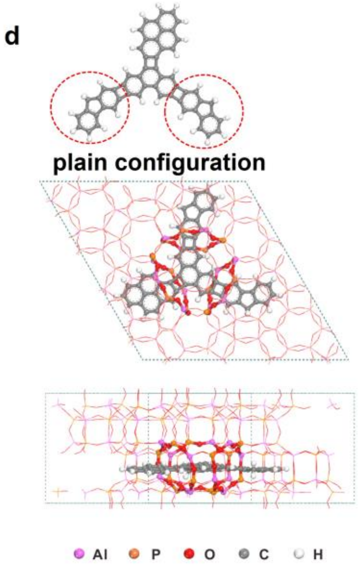

	kcal/mol
E_{inter}	-96.67
$E_{Strain\text{-}zeo}$	30.54
$E_{Strain\text{-}coke}$	26.33
E_{ads}	-39.80

Supplementary Fig. 9 The configurational optimization for our proposed Model 1: 3-D configuration (a) and plain configuration (b) and Model 2: 3-D configuration (c) and plain configuration (d) for cage-passing PAHs.

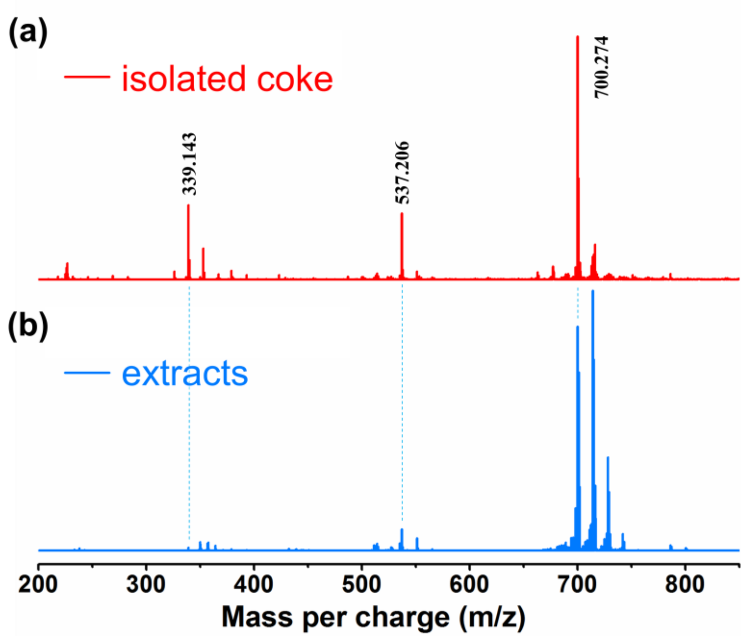

Supplementary Fig. 10 MALDI FT-ICR mass spectrum of the extracts by CCl_4 extraction (**a**) and isolated coke by suction filtration of the extracts (**b**) obtained from the spent SAPO-34 after 130 min MTO reaction.

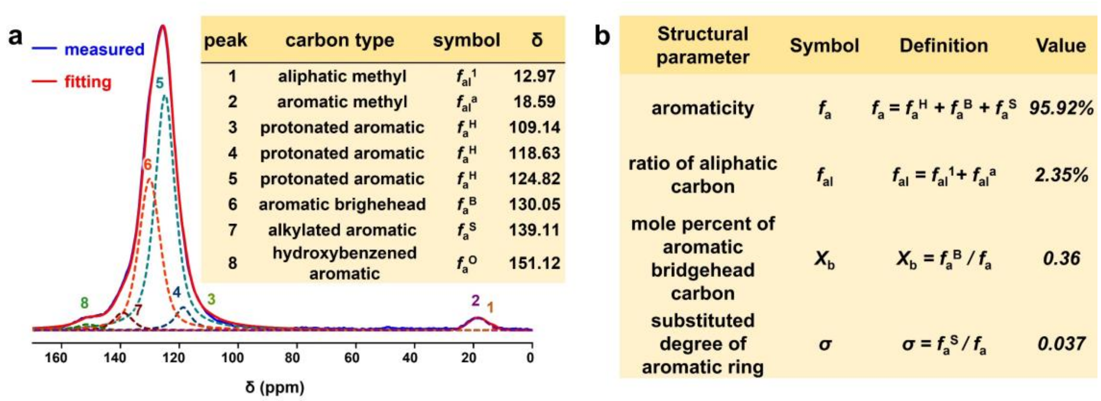

Supplementary Fig. 11 ^{13}C ssNMR spectrum of the isolated coke (which was obtained by suction filtration of the extracts released from the spent SAPO-34) with the assignments of the fitted eight peaks (**a**). The structural parameters and the calculated values (**b**).

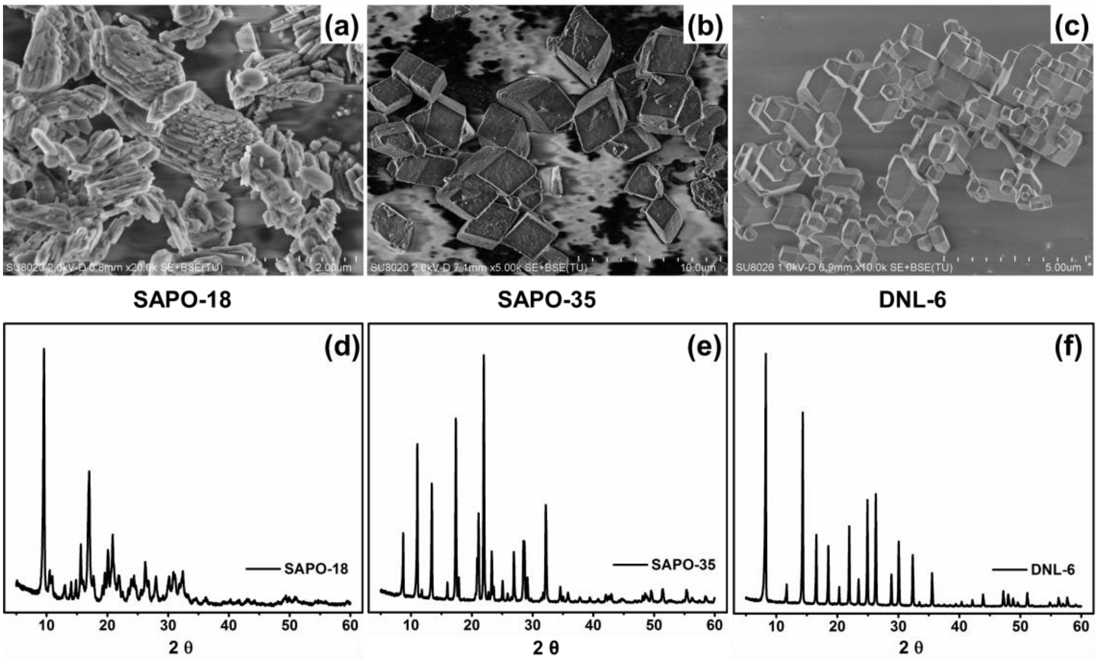

Supplementary Fig. 12 SEM images (upper) for SAPO-35 (a), SAPO-18 (b) and DNL-6 (c), and XRD patterns (bottom) for SAPO-35 (d), SAPO-18 (e) and DNL-6 (f).

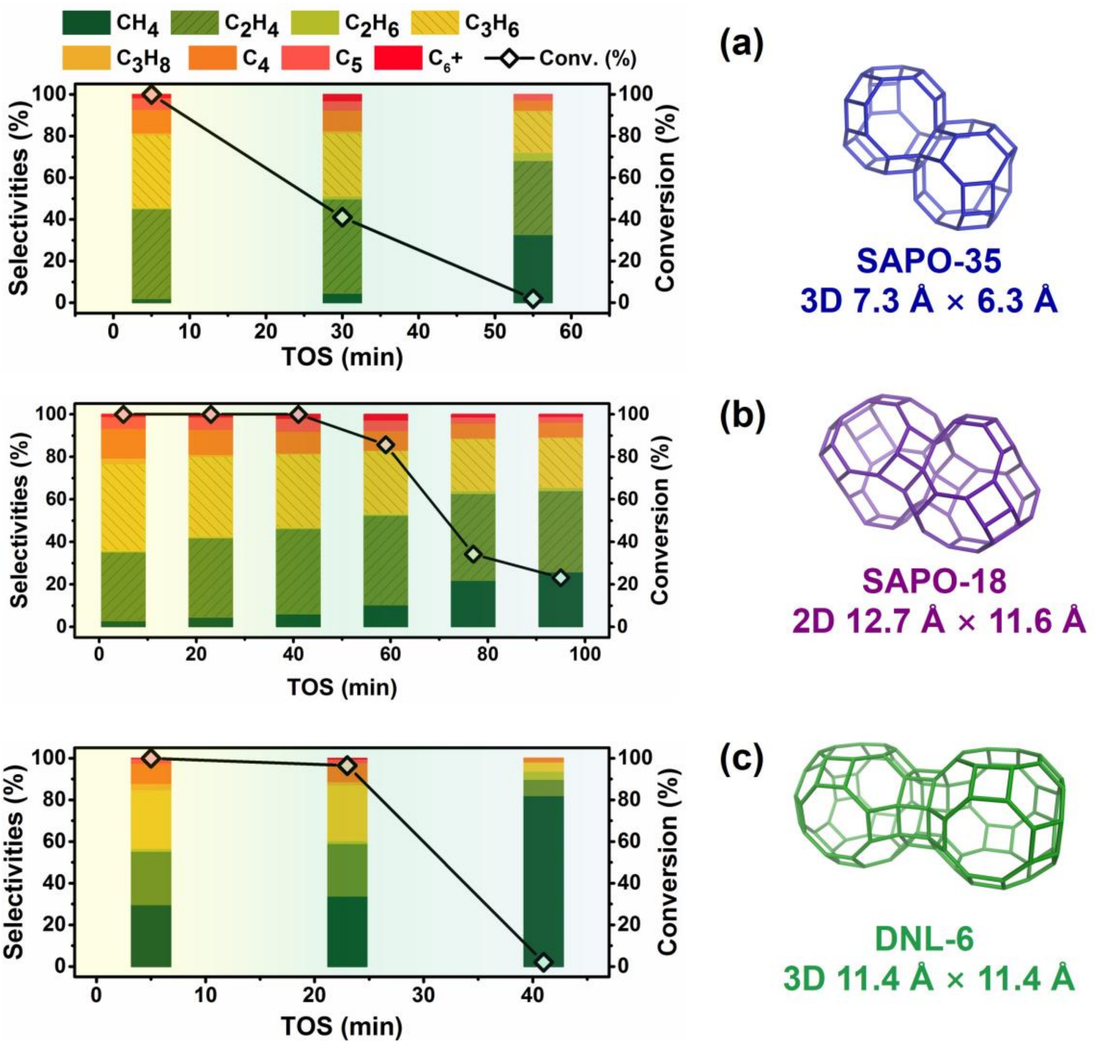

Supplementary Fig. 13 MTO reaction performances over SAPO-35 (a), SAPO-18 (b) and DNL-6 (c) at 475 °C and WHSV of 4 h^{-1}.

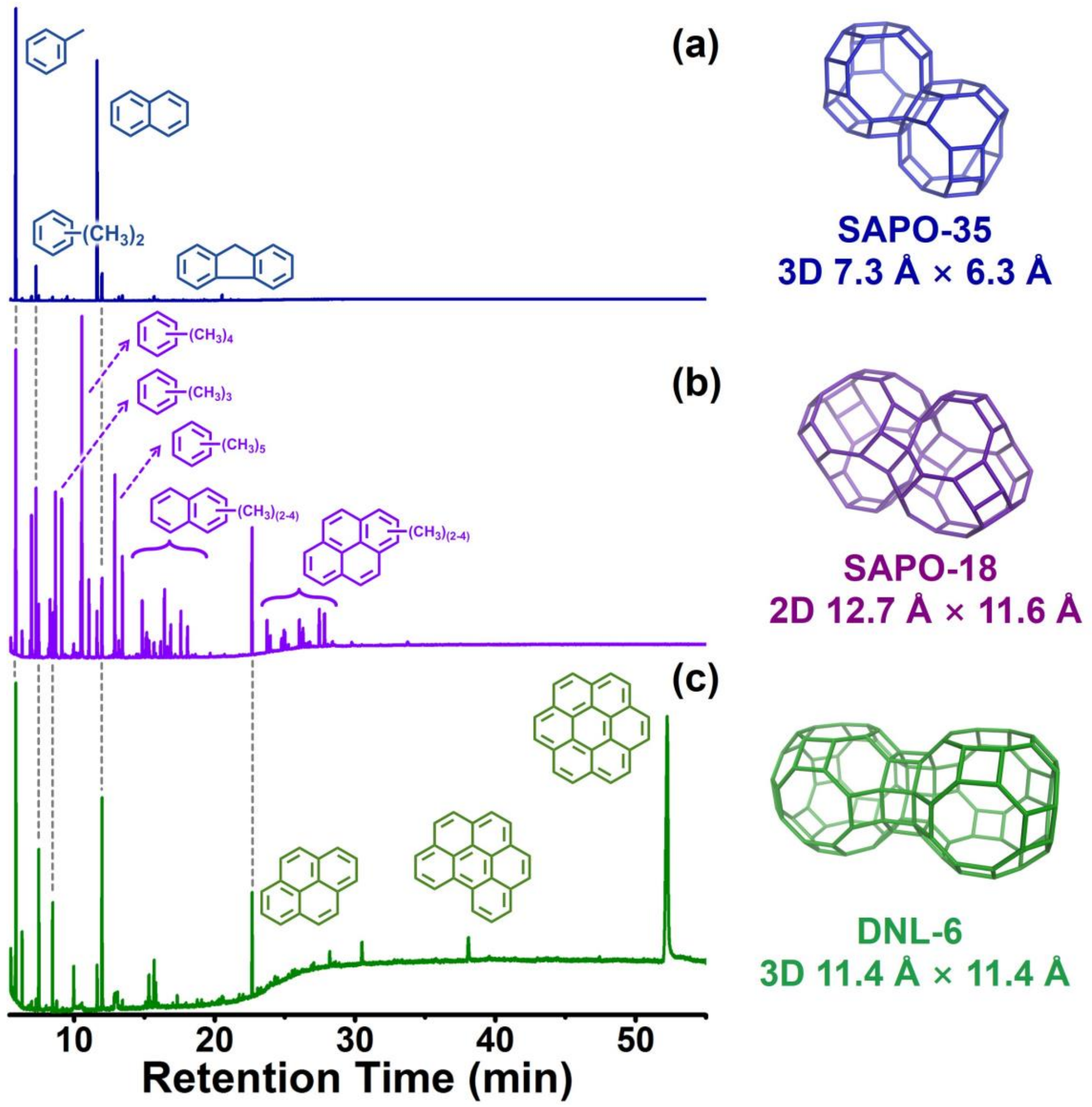

Supplementary Fig. 14 GC-MS analysis of CCl_4 soluble fractions of the retained organics (soluble "coke") extracted from the spent SAPO-35 (a), SAPO-18 (b) and DNL-6 (c) catalysts after HF digestion.

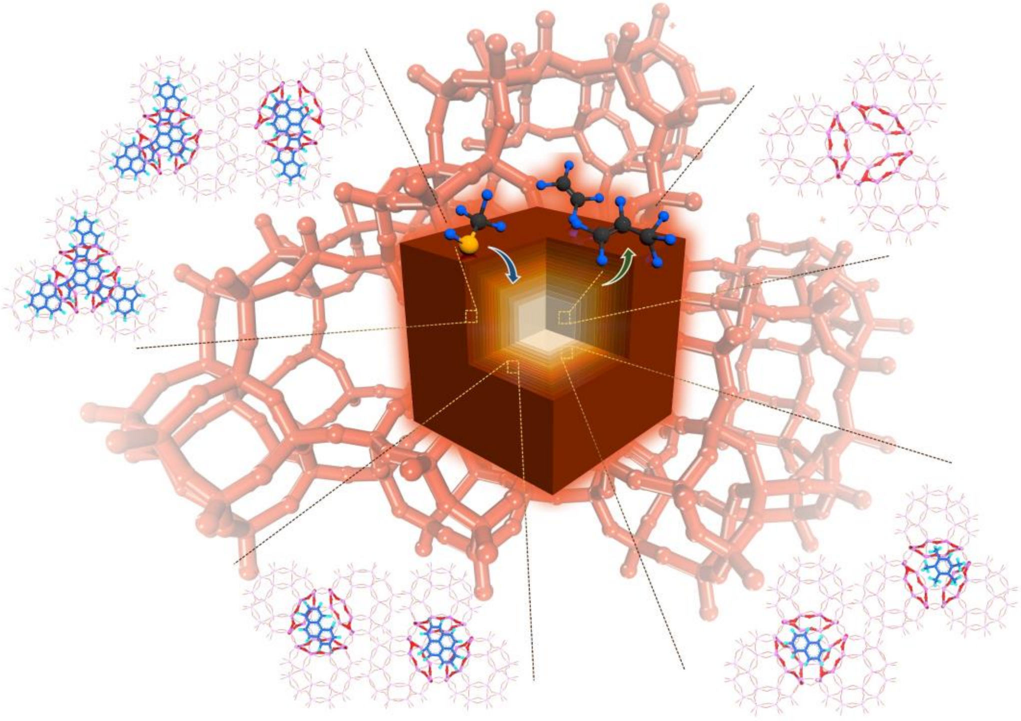

Supplementary Fig. 15 Proposal of a complete deactivating model. The model highlights the spatial distributions of a full-spectrum, well structurally determined “coke” entities, ranging from the imprisoned active aromatics (sited in the near-core part of crystal) to cross-linked, cage-passing grown heavier PAHs.

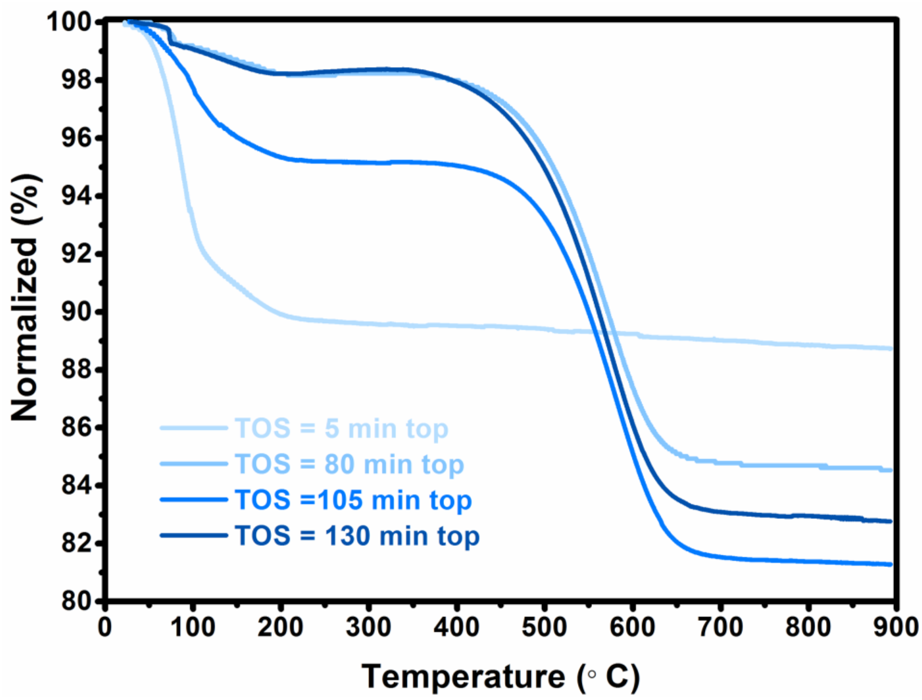

Supplementary Fig. 16 Normalized weight loss for the used SAPO-34 after MTO reaction for different durations at 475 °C and WHSV of 4 h^{-1}.

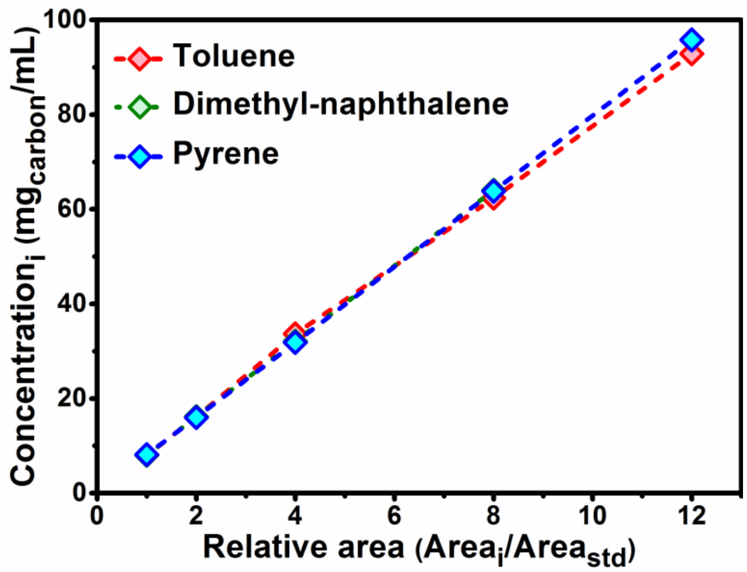

Supplementary Fig. 17 Calibration curves for different aromatic species. The integrated area of each compound in the chromatogram is normalized with the area of the internal standard (C_2Cl_6).

Supplementary Tables

Supplementary Table 1 Pore textural properties of parent SAPO-34 and used SAPO-34 after MTO reaction for different durations.

Samples	Surface area (m^2 g^{-1})			Pore volume (cm^3 g^{-1})	
	S_{BET}[a]	S_{Micro}[b]	S_{Exter}[b]	V_{Micro}[b]	V_{Meso}[c]
SAPO-34	516.6	510.0	6.6	0.251	0.010
SAPO-34-5 min	432.1	419.1	12.9	0.192	0.019
SAPO-34-55 min	96.0	88.0	8.0	0.041	0.008
SAPO-34-80 min	64.1	58.8	5.3	0.024	0.012
SAPO-34-130 min	44.2	40.6	3.6	0.019	0.003

[a]Total surface area is calculated by BET equation;

[b]Micropore surface area, external surface area, and micropore volume are determined by t-plot method;

[c]Mesopore volume is calculated by BJH method.

Supplementary Table 2 The elemental composition of SAPO-34 measured by XRF and XPS.

**Elemental composition (mol %)**		
**XRF**	**XPS**	**$Si_{surface}/Si_{bulk}$**
**$Si_{0.085}Al_{0.491}P_{0.424}O_2$**	**$Si_{0.100}Al_{0.480}P_{0.420}O_2$**	**1.2**

$Si_{surface}/Si_{bulk}$ = [Si/(Si+P+Al)] (by XPS) / [Si/(Si+P+Al)] (by XRF)

Supplementary Table 3 Molecular composition of the extracts and the examples of the possible molecular structures identified.

Family		A	B	C
Formula		C_nH_{2n-38} ($27 \leq n \leq 31$)	C_nH_{2n-64} ($43 \leq n \leq 48$)	C_nH_{2n-84} ($56 \leq n \leq 61$)
m/z range		340 - 396	538 - 608	700 - 770
UN[a]		20	33	43
Maximum peak		340 ($C_{27}H_{16}$)	538 ($C_{43}H_{22}$)	700 ($C_{56}H_{28}$)
Isotope shift	δ (^{13}C-^{12}C)[b]	27	43	56
	ΔC[c]	-	16	13
	δ (D-H)[d]	16	22	28
	ΔH[e]	-	6	6
Cages occupied		2	3	4

[a]Unsaturation number (UN).

[b]The carbon number difference (C_x) by comparing the respective maximum MS peaks between ^{13}C-methanol feed and ^{12}C-methanol feed.

[c]Carbon number of building units' difference by comparing the carbon number of each PAH with the preceding one.

[d]The hydrogen number difference (H_y) by comparing the respective maximum MS peaks between D-methanol feed and H-methanol feed.

[e]Hydrogen number of building units' difference by comparing the hydrogen number of each PAH with the preceding one.

Supplementary Table 4 The structural features and the acidity properties of SAPO-35, SAPO-18 and DNL-6.

Topology	AEI	LEV	RHO
**Cage dimensions (Å^2)**	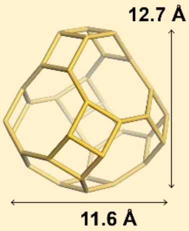	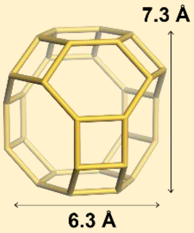	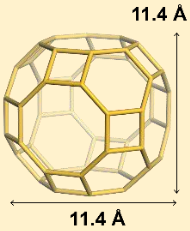
**Window dimensions (Å^2)**	**3.8 × 3.8**	**3.8 × 4.8**	**3.6 × 3.6**
**Pore structure (2-D/3-D)**	**3-D**	**2-D**	**3-D**
**Composition (measured with XRF)**	$Si_{0.122}Al_{0.487}P_{0.391}O_2$	$Si_{0.125}Al_{0.456}P_{0.419}O_2$	$Si_{0.148}Al_{0.477}P_{0.375}O_2$
**Acid sites per cage**	**1.5**	**1.3**	**3.6**

Supplementary Notes

Supplementary Note 1

Ruling out the possibility of appreciable on-surface coking. MALDI FT-ICR MS was directly performed to analyze the possible carbonaceous residues deposited on the external surface of used SAPO-34 with different reaction/deactivation extents (Supplementary Fig. 6). The experimental procedure was introduced in detail in Methods section in main text. For all three catalysts, mass spectra mainly showed the mass fragments in m/z of 227 and 451, which are assigned to dithranol and impurities, respectively. No obvious signals for coke species were detected. These observations further evidence the absence of appreciable external coking on our used SAPO-34.

Supplementary Note 2

Control experiments prior to carbonaceous deposit measurements. Power of incident laser influences the ionization severely. To determine an appropriate laser power—which should be strong enough to ionize the coke molecules yet not to polymerize or break them—to characterize the chemical structure of coke specie, we preliminary performed serious of control experiments using matrix (dithranol), model compound of 9,10-di-(2-naphthyl) anthracene with mass of 430 and extracted coke species from deactivated catalysts as standard or reference samples for testing under a gradient of laser power (Supplementary Fig. 7). For matrix, the laser output below 24% was safe to maintain the integrity of dithranol as shown in panel (**a**). For the used model compound, the upper limits of laser output for not inducing oligomerization was 24% (panel (**b**)), while this value was down to 18% for the extracted coke species (panel (**c**)). So, laser output of 18% was selected safely for the analysis of coke species.

Supplementary Note 3

Structural optimization of proposed cage-passing PAHs. The structural optimization of cage-passing PAHs with four possible conformations was further performed by the periodic density functional theory (DFT) calculation at PBE/DZVP level with the relaxing of the coke fragments and *cha* cages. The energetic estimations show that the strain energy of zeolite framework for Model 1a

is extremely high (105.88 kcal mol^{-1}) (Supplementary Fig. 9) and the framework deforms severely, which indicates that the cage-passing configuration of Model 1a is not so reasonable. While for Model 1b being structurally similar with Model 1a with 120 degree angles between each building block, the strain of the coke species and zeolite framework is significantly reduced to 30-50 kcal mol^{-1}, and adsorption energy is 20.79 kcal mol^{-1}. Considering the high temperature (up to 475 °C) for the coke formation, such deformation could possibly be acceptable.

As for Model 2a of cage-passing PAHs with 3-D configuration, the deformation of the coke species is relatively high ($E_{strain-coke}$ = 74.92 kcal mol^{-1}). While for Model 2b with plain configuration (with 120 degree angles between each building block), the deformation of coke species is much lower with the energy of 26.33 kcal mol^{-1}, and the adsorption energy is -39.8 kcal mol^{-1} much stronger than that of Model 2a with the energy of -7.12 kcal mol^{-1}.

Consequently, for both Model 1 and 2, the plain configurations of cage-passing PAHs (that is, Model 1b and Model 2b) are more energetically feasible.

Supplementary Note 4

The same mass distributions of the extracts with the isolated coke. Both the extracts by CCl_4 extraction and isolated coke by suction filtration of the extracts obtained from the spent SAPO-34 after 130 min MTO reaction were analyzed by MALDI FT-ICR MS using dithranol as matrix. The two mass spectra exhibited similar mass distributions, indicative of the identical nature of these two samples (Supplementary Fig. 10). That is, the isolated insoluble coke appearing as fine particles is suspended in the extraction liquid after HF dissolution-CCl_4 extraction. This also reflects the insoluble nature (in CCl_4) of extracts applied for MALDI FT-ICR MS. The above results validate the feasibility of taking isolated coke for further ^{13}C NMR structural analysis in Fig. 4c in the main text.
